# Carbon monoxide refines ovarian structure changes and attenuates oxidative stress via modulating of heme oxygenase system in a rat model of polycystic ovary syndrome: An experimental study

**DOI:** 10.18502/ijrm.v22i8.17231

**Published:** 2024-10-14

**Authors:** Bahareh Asadi, Kamran Rakhshan, Mina Ranjbaran, Arash Abdi, Maryam Vaziripour, Behjat Seifi

**Affiliations:** Physiology Department, School of Medicine, Tehran University of Medical Sciences, Tehran, Iran.

**Keywords:** PCOS, Rat, Oxidative stress, Heme oxygenase, CORM.

## Abstract

**Background:**

Carbon monoxide (CO), influences ovarian function, pregnancy, and placental health. Heme oxygenase (HO)-1 and its products, including CO, exhibit protective and anti-inflammatory properties.

**Objective:**

This study investigates the protective effects of CO released by the carbon dioxide-releasing molecule (CORM)-2 against oxidative stress, functional and structural changes of the ovaries, and HO-1 expressions in female rats suffering from polycystic ovary syndrome (PCOS).

**Materials and Methods:**

In this experimental study, 24 *Rattus norvegicus var. Albinus* female rats (180–200 gr, 8 wk) were randomly divided into 4 groups (n = 6/each): control, CORM-2 (10 mg/kg), PCOS (induced by 4 mg/kg, intramuscular injection and a single dose of estradiol valerate), PCOS + CORM-2. Ovary histological changes were evaluated by crystal violet staining. Malondialdehyde (MDA) level and superoxide dismutase (SOD) activity of ovarian tissue were assessed using enzyme-linked immunosorbent assay. HO-1 expression was evaluated using Western blot.

**Results:**

Corpus luteal formation significantly decreased in the PCOS group and was significantly restored with CORM-2 administration compared to the control group (p < 0.05). The expression of ovarian HO-1 protein was reduced in the PCOS group compared to controls (p < 0.01), and administration of CORM in PCOS rats significantly increased its expression (p < 0.0001). In addition, CORM administration markedly reduced ovarian MDA levels and restored SOD activity (p < 0.0001).

**Conclusion:**

CORM-2 administration to PCOS rats created protective effects by reducing oxidative stress (reducing MDA level and restoring SOD activity) and increasing ovarian HO-1 protein.

## 1. Introduction

Polycystic ovary syndrome (PCOS) is a frequently occurring hormonal condition among women of childbearing age, affecting approximately 48% of this demographic. Despite extensive research, the pathophysiology of PCOS is not yet fully understood. So far, the role of inflammatory factors, endothelial damage, and oxidative stress (OS) has been partially identified in PCOS pathophysiology and treatment.

In the not-so-distant past, oxygen (O_2_) was the only important gas among all gas molecules, while today, it is important to consider gas transporters such as hydrogen sulfide, nitric oxide, and carbon monoxide (CO) to regulate physiological functions. Regulation of inflammatory responses in female reproductive systems is just one of the most important roles of these 3 gaseous molecules (1). These gas molecules sometimes interact with each other; in the case of hydrogen sulfide and nitric oxide, these gas transporters are important for maturation and completion of meiosis after ovulation (2).

The presence of CO in the ovaries, uterus, and placenta has been proven (3). In humans, physiologically, the rate of CO production is 16.4 µm/hr, and its daily production reaches 500 µm. This low-concentration gas carrier has several physiological functions, such as vasodilation, neurotransmission, inhibition of platelet aggregation, cellular protection, and antiproliferative effects (4). Other studies have shown that CO plays an important role in regulating ovarian function and pregnancy through proper placental function during pregnancy, steroidogenesis and corpus luteum survival (5, 6). The protective, anti-inflammatory, and anti-apoptotic functions of heme oxygenase (HO)-1 and its products, including CO, have also been demonstrated.

The novelty of this research article lies in its investigation of the protective effects of CO released by the carbon dioxide-releasing molecule (CORM)-2 in female rats suffering from PCOS. Both endogenous and exogenous CO, CORMs, can increase gene expression and phosphorylation of transcription factors and antioxidant elements such as superoxide dismutase (SOD); also affect oxidant signals by regulating antioxidant signaling pathways (7). HO-1 and 2, on the other hand, are expressed in Teca cells, granulosa cells of follicles, and luteal cells in the ovaries of adult mouse, resulting in the expression of stressors (8). This enzyme is also expressed in ovarian stroma (9). Impaired HO/CO system leads to reproductive failure (10). Therefore, due to the importance of HO/CO in ovulation and PCOS, there is a need to focus on this reproductive process (11).

Today, supportive methods of weight loss, proper diet, exercise, and ovulation-inducing drugs such as contraceptives, surgery, and finally, in vitro fertilization techniques are used to induce pregnancy in PCOS women. Ovulation-inducing drugs and surgery are not fully effective. In vitro fertilization methods lead to twin or multiple pregnancies, increased ovarian cysts, and increased risk of ovarian cancer (12). In addition, the presence of oxidative factors in serum and follicular fluid and improper mitochondrial function in granulosa cells of PCOS women weaken the effect of in vitro fertilization methods (13).

Therefore, it seems necessary to find new solutions due to the complete inefficiency of these methods. In order to suggest an appropriate treatment, this study aims to investigate the protective effects of CO released by the CORM-2 on OS, functional and structural changes of the ovaries, and the amount of HO-1 expression in female rats suffering from PCOS.

## 2. Materials and Methods

### Animals

In this experimental study, which was conducted at Tehran University of Medical Sciences, Tehran, Iran between 2022 and 2023, 24 female *Rattus norvegicus var. Albinus* rats (180–200 gr, 8 wk) were enrolled. The animals were kept in standard conditions in terms of light (12 hr light/ dark cycle), 22 ± 2 C and humidity of 30–40% during the protocol. The animals were randomly divided into 4 groups (n = 6/each): 1) control, 2) CORM-2, 3) PCOS, and 4) PCOS + CORM-2 group. In all rats, a vaginal smear was performed for 6 days before PCOS induction to determine the estrous cycle by light microscopy and stained with crystal violet (Tebvaran, Iran). If there was any 1 cycle, then pro-estrus, estrus, met-estrus, and di-estrus rats were included in the study (Table I).

It should be noted that since the sexual cycle in PCOS rats is disturbed and stops in one phase of the estrous cycle, control rats in the estrus phase were used in all stages of the work to eliminate the effect of the sexual cycle on the test results.

The studied animals were anesthetized before sampling by intraperitoneal injection of ketamine (100 mg/kg, ketamine hydrochloride^TM^, Rotexmedica, USA) and xylazine (10 mg/kg, xylazine hydrochloride^TM^, Sigma, USA), then placed in the supine position, followed by a vertical midline incision in their abdomen. At the time of sampling, the ovaries of the animals were carefully removed from the body and washed in normal saline to drain the blood. Then excess fat was carefully removed without any damage to the ovarian tissue, to determine the weight and volume of the ovaries.

For rapid freezing, the left ovaries were immediately placed in an aluminum foil and immersed in liquid nitrogen, then placed in a freezer at -70 C for analysis. Left ovaries were used to evaluate OS factors as well as the amount of OH-1 protein in ovarian tissue. The right ovaries were placed in a 10% formalin fixation solution (Merc, Germany) for histological examination.

In the PCOS group, after evaluating the estrus cycle for 6 days, IM injection and a single dose of estradiol valerat (Aburaihan [NOVO Nordisc], Iran), 4 mg/kg + 0.2 ml sesame oil (Adonis Daru, Iran) were administered to induce the polycystic ovary model. In PCOS + CORM-2 (RU2cl2 Tricarbonyldichlororuthenium 2^TM^, Sigma, USA) group, CORM-2 (10 mg/kg) was injected intraperitoneally daily from day 22–31 (14).

To assess the condition of the ovaries in all 4 groups, sampling was performed on the 35 ${\;}^{\text{th}}$ day. A vaginal smear was prepared for 3 days to confirm the induction of the model and the cessation of the estrus cycle. Histological examinations were also used to confirm stopping the follicular cycle by the pre-antral stage (2).

### Evaluation of the sexual cycle (estrus cycle)

A vaginal smear (vaginal swab) was collected every 6^th^ day before induction, and the sexual cycle was determined. The main stages consist of pro-estrus, estrus, met-estrus and di-estrus. To determine the estrous cycle stages, epithelial cells were stained by crystal violet staining and examined under a light microscope (15).

### Preparing vaginal smear

First, the mouse was placed in a supine position where the vaginal opening was up and accessible. Then 100 µL of sterilized deionized water (ddH ${\;}_{2}$ O) were slowly drained through the sampler head into the vaginal canal, and after 10 sec the same fluid was returned to the sampler head, this process was repeated 3 times. To prevent any false pregnancy in the rats, hitting the vagina with a sampler head was protected (16).

### Vaginal smear cytological staining by crystal violet

To prepare 0.1% crystal violet dye solution, 0.1 gr of violet crystal powder was dissolved in 100 ml ddH ${\;}_{2}$ O. The slide containing the dried vaginal smear was then placed in a glass container containing a crystal violet solution for 1 min. The slide was then removed from the dye solution and washed twice at ddH ${\;}_{2}$ O for 1 min each time. After washing, excess ddH ${\;}_{2}$ O was removed from the edges of the slide, and 15 μl of glycerol (Merck, Germany) was poured onto the smear. Then, a lamellar (ExtraGene, Taiwan) was placed on it, and a light microscope was used for cell studies (17).

### Morphological study of the ovary

To evaluate the effect of estradiol valerate on ovarian cyst formation, 1 ovary of each group was fixed in a 10% fixative solution with pH = 7.2 for 72 hr and molded in paraffin after dewatering. Serial 5 μm-thickness incisions of the ovaries were prepared and stained with hematoxylin-eosin. Digital images of these sections were captured using a microscope equipped with a digital camera. Using ImageJ software, follicle diameters, corpora lutea (CL) area, antral cavity sizes, and the thickness of ovarian layers were assessed to distinguish the types of follicles in the counting process. Cystic follicles, identified by their larger size and thickened granulosa and theca layers, were specifically examined for fluid-filled spaces. The area and morphological details of CL, including the presence of blood vessels and connective tissue, were also evaluated. Measurements from 5 randomly selected sections were collected per animal to ensure accuracy, and statistical analyses were performed to compare findings between groups (18).

### Measurement of malondialdehyde (MDA) and SOD in ovarian tissue

MDA was measured using the Esterbauer and Cheeseman method and based on its reaction with thiobarbituric acid (19). Ovarian tissue (50 mg) was homogenized with 10% trichloroacetic acid (TCATM, Sigma, USA), followed by centrifugation to precipitate proteins. The resulting solution underwent a reaction with 0.67% thiobarbituric acid (TBATM, Sigma, USA), was heated, and then its adsorption at 532 nm was measured. The concentration was calculated based on a standard curve. SOD enzyme activity was determined using an ELISA kit (Navid Salamat CO., Iran). For each 100 mg of ovarian tissue, lysis buffer was added and the resulting solution was centrifuged at 1 C. The supernatant was then used for measuring SOD activity with the kit (20).

### Measurement of HO-1 activity in ovarian tissue using Western blot technique

The Western blot analyses were carried out according to the previously described protocol, with some modifications (9). The lysates were removed, after being subjected to centrifugation at 14,000 rpm for 20 min at 4 C. The lysate's protein concentration was determined using the bicinchoninic acid protein Quantification kit, following the manufacturer's instructions. The exosome lysates were mixed with an equal volume of 2X Laemmli sample buffer to prepare the samples. Lysates (15 μg) were loaded onto an SDS-PAGE gel, boiled for 5 min, and transferred onto a 0.2 μm polyvinylidene flouride membrane using Immune-Blot ${\;}^{\text{TM}}$ from Bio-Rad Laboratories (CA, USA). The unspecific interactions were blocked with 5% BSA in 0.1% tween 20 for 1 hr, after which they were washed 3 times with phosphate buffered saline. The membranes were then incubated with anti-HO-1 (Abcam) and anti-beta actin-loading control antibodies (Abcam) for 1 hr at room temperature. After being washed for 3 times with tris-buffered saline, the membranes were incubated with goat anti-rabbit immunoglobulin G H/L (Abcam) secondary antibodies and subsequently treated with enhanced chemiluminescence for 1–2 min. Protein expression was normalized to β-actin. The densitometry of protein bands was analyzed using NIH's gel analyzer software (version 2010a) (21).

**Table 1:** Intervention and evaluation procedures in experimental groups


**Group**	**Intervention**	**Duration**	**Key procedures**
**Control**	-	35 days	Vaginal smear for 6 days prior to PCOS induction
**CORM-2**	CORM-2 (10 mg/kg) intraperitoneal daily (days 22–31)	35 days	Ovary sampling for histology and biochemical analysis
**PCOS**	Estradiol valerate (4 mg/kg) IM injection	31 days	Vaginal smear for confirmation of PCOS induction
**PCOS+CORM-2**	Estradiol valerate (4 mg/kg) + CORM-2 (10 mg/kg) daily	35 days	Ovary sampling for histology, biochemical, and Western blot
CORM: Carbon dioxide-releasing molecule, PCOS: Polycystic ovary syndrome, IM: Intramuscular injection			

### Ethical considerations 

All experimental procedures and protocols were approved by the Animal Ethics Committee of Tehran University Medical Sciences, Tehran, Iran (Code: IR.TUMS.MEDICINE.REC.1400.957) with the Guide for the Care and Use of Laboratory Animals published by the United States National Institutes of Health (NIH Publication, 8 ${\;}^{\text{th}}$ Edition, 2011).

### Statistical analysis

GraphPad Prism 9.0 statistical software (GraphPad Software Inc., CA, USA) was applied for data analysis. Quantitative data were expressed as mean ± SEM and qualitative data as percentages. At first normal distribution of the data was investigated using normality tests (Kolmogorov-Smirnov method). Then the statistical analysis for quantitative variables was performed by one-way ANOVA followed by Turkey's post hoc to compare differences between 3 groups or more. Statistical levels below 0.05 were considered significant.

## 3. Results

### Estrus cycles in rats

The success of PCOS induction, 31 days after a single dose and IM injection of estradiol valerate was biochemically and histologically confirmed using crystal violet staining. PCOS rats were stopped in an estrus cycle after induction of disease, while controls had a normal rotational cycle of pro-estrus, estrus, met-estrus, and di-estrus.

### Effects of CORM-2 administration on CL, cystic follicles, preantral follicles, and preovulatory follicles

**Figure 1:**
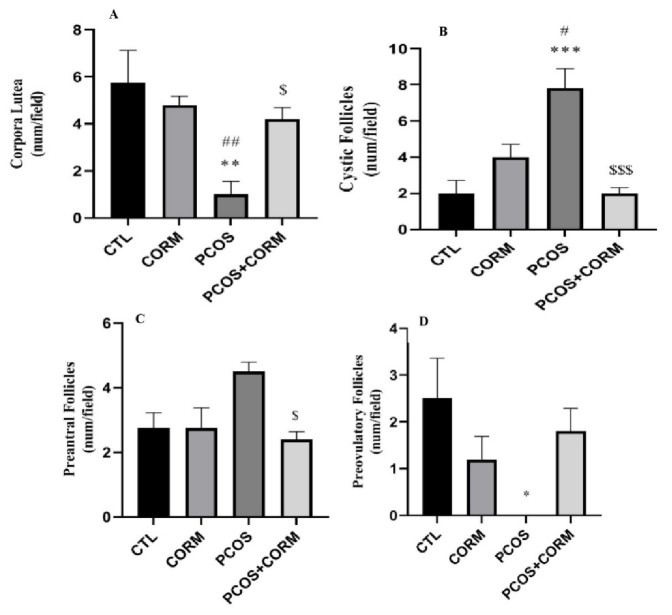
A) Corpora lutea, B) Cystic follicles, C) Pre-antral follicles, and D) Preovulatory follicles numbers at day 35 after developing the model. *P < 0.05, **P < 0.01, and ***P < 0.001 vs. control group, ${\;}^{\#}$ P < 0.05, ${\;}^{\#\#}$ P < 0.01 vs. CORM group and ${\;}^{\$}$ P < 0.05, ${\;}^{\$\$\$}$ P < 0.001 vs. polycystic ovarian syndrome (PCOS) group. CTL: Control, CORM: Carbon dioxide-releasing molecule, PCOS: Polycystic ovary syndrome.

Figure 1 shows the number of ovarian cystics and different types of follicles in all groups. The number of corpus lutea (CL) formed in the ovary was significantly decreased in the PCOS group compared to the control and CORM groups. Our results indicated that treatment with CORM-2 markedly restored the number of CL compared to the PCOS group (p < 0.05, Figure 1A). Figure 1B shows the number of formed cystic follicles. Compared to the control and CORM groups, the number of formed cystic follicles significantly increased in the PCOS group. Our results showed that CORM-2 administration markedly reduced the number of formed cystic follicles compared to the PCOS group (p < 0.001). Similar to the number of formed cystic follicles, the amount of pre-antral follicles increased in the PCOS group in comparison with the control and CORM groups (Figure 1C). A significant decrease in the amount of pre-antral follicles was observed in the PCOS + CORM-2 group (p < 0.05) than in the PCOS group. The number of preovulatory follicles was significantly decreased in PCOS compared to control (Figure 1D). Although CORM-2 administration in the PCOS + CORM-2 group increased preovulatory follicle formations more than in PCOS rats, it was not significant.

### Western blot of HO-1 enzyme activity in ovarian tissue

**Figure 2:**
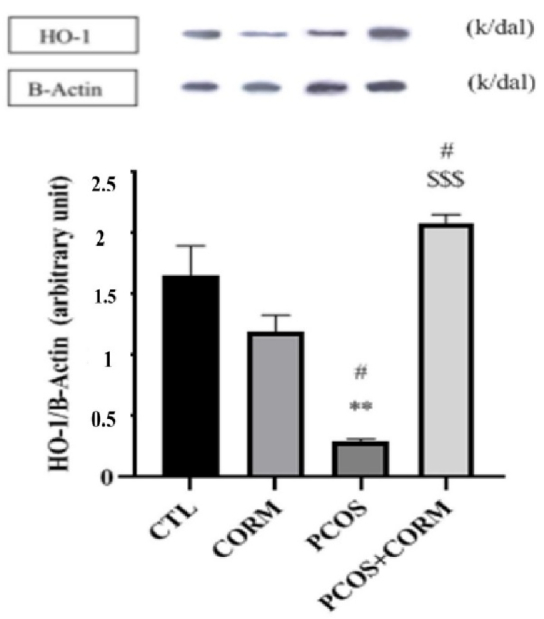
At day 35 after inducing the PCOS model, the normalized expression of HO relative to β-actin was assessed in different experimental groups. The results are presented as mean ± SEM. Statistical analysis revealed significant differences between groups (**P < 0.01 vs. control group, ${\;}^{\#}$ P < 0.05 vs. CORM group, ${\;}^{\$\$\$}$ P < 0.001 vs. PCOS group). HO: Heme oxygenase, CTL: Control, CORM: Carbon dioxide-releasing molecule, PCOS: Polycystic ovary syndrome.

Western blot showed that protein levels of HO as enzyme producing CO was significantly decreased in the PCOS group than in the control and CORM group (p < 0.01). CORM administration markedly increased protein levels of HO compared to the PCOS group (p < 0.001) (Figure 2).

### OS status (MDA and SOD)

**Figure 3:**
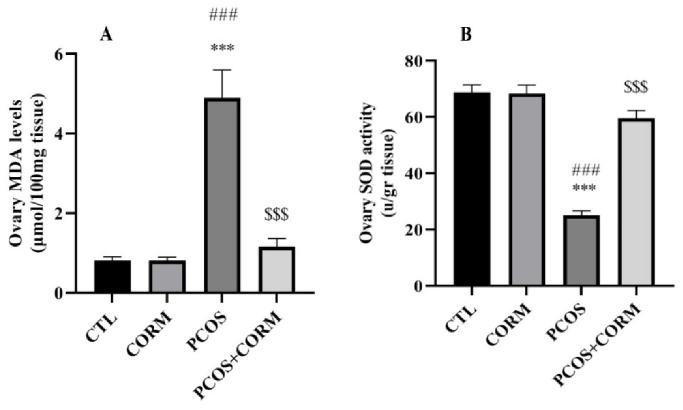
A) MDA, B) SOD levels at day 35 after induction of the PCOS model. ***P < 0.001 vs. control group, ${\;}^{\#\#\#}$ P < 0.001 vs. CORM group, ${\;}^{\$\$\$}$ P < 0.001 vs. PCOS group. MDA: Malondialdehyde, SOD: Superoxide dismutase, CTL: Control, CORM: Carbon dioxide-releasing molecule, PCOS: Polycystic ovary syndrome.

Figure 3 shows ovary MDA levels and SOD activity contributed to all groups. As depicted in figure 3A, MDA level, as an OS damage indicator, was markedly elevated in the PCOS group than in the control and CORM-2 groups (p < 0.001). A significantly reduced level of MDA was seen after the administration of CORM-2 as an antioxidant factor compared to the PCOS group (p < 0.001). In figure 3B, SOD activity was markedly reduced in PCOS group than control and CORM groups (p < 0.001). A significant recovery of SOD activity was seen in PCOS + CORM group compared to PCOS group (p < 0.001).

### Ovarian structural changes

**Figure 4:**
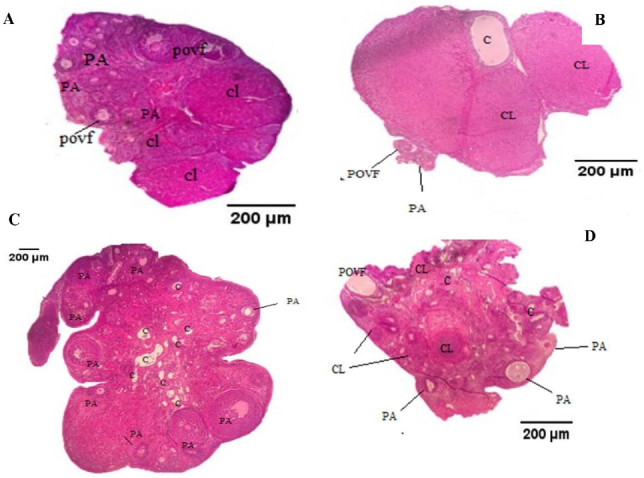
5 μm ovarian tissue sections stained with hematoxylin and eosin (H&E). Cross-sectional micrographs of the ovarian tissues ( × 400 magnified) show A) Control, B) CORM, C) PCOS, and D) PCOS+CORM groups. PA: Preantral follicles, Povf: Preovulatory follicles, CL: Corpora lutea, and C: Cystic follicles.

Qualitative histological analyses, conducted by 2 blinded and experienced pathologists, revealed that PCOS rats exhibited a higher pre-antral follicle distribution compared to the control and CORM groups. Furthermore, the findings indicated a decrease in the distribution of pre-antral follicles and an increase in the distribution of preovulatory follicles and CL in the PCOS + CORM group compared to the PCOS group (Figure 4).

## 4. Discussion

PCOS humans and animals exhibit an increase of OS factors besides decreasing antioxidant agents in the ovaries. In the present study, the status of OS and action of CORM-2 administration as an antioxidant factor was evaluated in an animal model. We showed that CORM-2 in PCOS rats not only decreased ovarian OS by reducing MDA and increasing SOD activity but also increased CO by elevating HO/β-actin in the ovarian tissue western blot samples. Additionally, we noticed lowering numbers of histologically cystic follicles after CORM-2 administration to PCOS rats.

As mentioned, PCOS is one of the most prevalent endocrinological pathologies involving ladies at some stage in their reproductive years displaying an extensive spectrum of scientific manifestations. PCOS sufferers have been shown to have an-ovulatory infertility. On the other hand, OS is generally referred to how there is an imbalance among oxidants and antioxidants. When this imbalance turns in favor of oxidants, reactive oxygen species (ROS) are released, which damage the body in several ways with a huge generation of such ROSs. With other aspects, reproductive tissues and cells will continue to be in a stable condition if oxidants and antioxidants are in balance. OS, as a generally known factor in PCOS women, regardless of the presence of metabolic abnormalities, is present in infertile women. Reactive and unstable free radicals lead to cell destruction by stealing electrons from carbohydrates, proteins, nucleic acids, lipids, and other nearby molecules. 2 major types of free radicals are ROS and reactive nitrogen species. ROS are O2 centers surrounded by free radicals. These are unstable structures with an outer covered layer of unpaired electrons, which tend to react with other radicals and molecules achieving stable configuration by pairing electrons (22).

Various primitive cellular reactions result in the production of ROS inside the cells. In the electron transport chain, molecular O2 is converted to water (H ${\;}_{2}$ O) during the cellular respiration process. It has been seen that a chain of reactions contribute to cells O ${\;}_{2}$ molecules deduction and production of hydrogen peroxide (H ${\;}_{2}$ O ${\;}_{2}$ ), superoxide anion radical and the hydroxyl radical as 3 major ROS species: (i) O ${\;}_{2}$ + e ${\;}^{-}$ → O ${\;}_{2}{\;}^{-}$ , (ii) 2O ${\;}_{2}{\;}^{-\cdot }$ + 2H ${\;}_{2}$ O → H ${\;}_{2}$ O ${\;}_{2}$ + O ${\;}_{2}$ , (iii) O ${\;}_{2}{\;}^{-\cdot }$ + H ${\;}_{2}$ O ${\;}_{2}$ → OH + OH ${\;}^{-}$ + O ${\;}_{2}$ (23).

Also, it has been declared that by using mitochondrial SOD 2, superoxide radicals will be converted to hydrogen peroxides and after modification by glutathione peroxidase, will form water in the cell. They confirmed that the presence of antioxidant agents are vital for maintaining cell redox hemostasis, and its absence leads to cell destruction. In the present study, we discovered that CORM-2 administration to PCOS rats could increase SOD as an antioxidant factor to improve ovary condition and status of the disease (24).

CORM-2 releases CO in a concentration-based condition, which is modulated through pH, temperature, and environment (25). CO synthesis is a result of HO-1 activity during the heme degradation process. HO-1, by converting prooxidant heme to biliverdin and then bilirubin (26), releases CO as a side product (26). Some studies demonstrated the beneficial physiological effects of CO on vascular and inflammatory diseases (27). Also, some suggest that CO may have neuroprotective effects, meaning it could help protect nerve cells from damage (28). Additionally, it was discovered that HO-1 and its bioactive metabolite CO could be effective in downregulating major hypoxia-induced factors such as OS (29–31). In the present study, CORM-2 administration to PCOS rats significantly increases HO-1 in ovarian tissue. Another interesting finding was that HO/β-actin decreases in the CORM-2 group than control ones. Although this difference is not significant, CORM-2, as an exogenous emancipator of CO, could lead to decreasing HO-1 (the enzyme producing CO) in physiologic circumstances. This might be attributed to toxic levels of CO administration as an exogenous agent in healthy rats. Moreover, comparing CORM-2 and PCOS + CORM-2 groups showed a significant increase of HO/β-actin in latter than the CORM-2 group. Since the need of cells for CO is little in healthy conditions and HO levels are within normal limits in physiological conditions, CORM-2 administration may lead to lower production of HO-1 in normal cells. However, in pathological OS conditions in which HO levels, generally tends to decrease, administration of CORM-2 may elevate HO-1 enzyme concentrations.

On the other aspect, reviewing the structure of the ovary in the present study showed CORM-2 increased CL formation approximately the same as the control group. It is a fact that CO has toxic effects at certain concentrations, and the addition of CORM-2 as an external agent in healthy rats may increase the level of CO to a toxic level and reduce the growth of CL slightly. However, CORM-2 administration in PCOS rats might increase CO concentration to the physiologic levels, which could be effective in increasing the growth of CL formations. Moreover, in the CORM-2 group, the number of formed cystic follicles in normal animals are higher than in controls, which also mentioned toxic levels of CO by adding CORM-2 as an external factor. In addition, the quantity of preovulatory follicles in the CORM-2 group is also lower than in control group. Till now, there has not been enough study on the effect of CORM-2 on ovary structural changes and estrous cycling, and the present study is the first to report the CO effect on PCOS. In a study, curcumin administration to PCOS rats caused a re-evaluation of ovarian structure. They reported different types of ovarian follicles in PCOS rats (30, 31). They found decreases in differentiation of ovarian follicles (32). Despite this study in which all follicular clusters were found to be the most frequent in the PCOS group than others, in our study, CL and preovulatory follicles in PCOS rats were at the least quantity compared to others.

## 5. Conclusion

In summary, CORM administration to PCOS rats improved ovarian structural changes by reducing OS and restoration of the endogenous antioxidant system. Moreover, our findings showed that the protective effects of CORM administration in PCOS rats were associated with increasing HO-1 protein expression.

##  Data availability

Data is available from the corresponding author upon request.

##  Author contributions

B. Seifi and B. Asadi designed the study and conducted the research. B. Seifi, K. Rakhshan, and M. Ranjbaran monitored, evaluated, and analyzed the results of the study. A. Abdi and M. Vaziripoor helped in the experimental stages. Further, all authors reviewed the article. All authors approved the final manuscript and take responsibility for the integrity of the data.

##  Conflict of Interest

All of the authors declare no conflict of interest.
